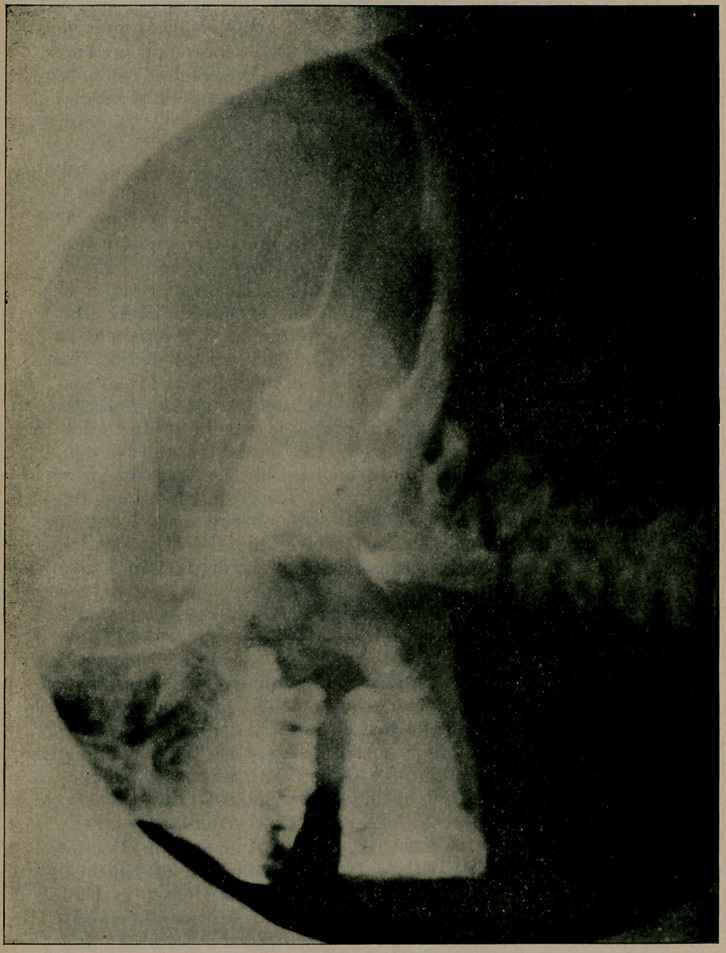# Non-Fatal Fracture of Axis

**Published:** 1916-03

**Authors:** 


**Non-Fatal Fracture of Axis.** F. W. O'Brien, Boston, Boston M. & S. Jour., Jan. 20, 1916. (Cut by courtesy of Editor). Corner, Annals of Surg., 1907, collected 8 fatal cases in 6 of which the odontoid was broken, and 10 non-fatal cases in only one of which the odontoid was broken. Mixter and Osgood, Am. Jour. Orthopaedics, Feb. 1910, review the literature of traumatic lesions of the upper two cervical vertebrae, cal special attention to the abundance of space about the cord in this region, explain on this basis, the lack of symptoms in non-fatal cases, and emphasize the marked tendency to death when the odontoid is broken. (See also abstract of report of dislocation of atlas, with illustrations, page 390-391, of our

Feb. issue). O'Brien's case was that of a boy aged 7, who had fallen from a tree on the back of his head five days before he was consulted. The complaint was of inability to turn the head without pain, and the child supported the ramus of the jaw with his hand. Otherwise there were practically no symptoms, none at all referable to cord paralysis. There was complete fracture of laminae of axis as shown. Dr. G. A. Moore applied an orthopaedic support. This was removed after five weeks, the second radiogram showing excellent union, in fact the fracture line was scarcely visible.

## Figures and Tables

**Figure f1:**